# Targeting of a Chlamydial Protease Impedes Intracellular Bacterial Growth

**DOI:** 10.1371/journal.ppat.1002283

**Published:** 2011-09-29

**Authors:** Jan G. Christian, Julia Heymann, Stefan A. Paschen, Juliane Vier, Linda Schauenburg, Jan Rupp, Thomas F. Meyer, Georg Häcker, Dagmar Heuer

**Affiliations:** 1 Institute for Medical Microbiology, Immunology and Hygiene, Technische Universität München, Munich, Germany; 2 Institute for Medical Microbiology and Hygiene, University Hospital Freiburg, Freiburg, Germany; 3 Max Planck Institute for Infection Biology, Dept. Molecular Biology, Berlin, Germany; 4 Robert Koch-Institute, Junior Research Group 5, Berlin, Germany; 5 Institute of Medical Microbiology and Hygiene, University of Lübeck, Lübeck, Germany; 6 Medical Clinic III, UK-SH/Campus Lübeck, Lübeck, Germany; Institut Pasteur, France

## Abstract

Chlamydiae are obligate intracellular bacteria that propagate in a cytosolic vacuole. Recent work has shown that growth of *Chlamydia* induces the fragmentation of the Golgi apparatus (GA) into ministacks, which facilitates the acquisition of host lipids into the growing inclusion. GA fragmentation results from infection-associated cleavage of the integral GA protein, golgin-84. Golgin-84-cleavage, GA fragmentation and growth of *Chlamydia trachomatis* can be blocked by the peptide inhibitor WEHD-fmk. Here we identify the bacterial protease chlamydial protease-like activity factor (CPAF) as the factor mediating cleavage of golgin-84 and as the target of WEHD-fmk-inhibition. WEHD-fmk blocked cleavage of golgin-84 as well as cleavage of known CPAF targets during infection with *C. trachomatis* and *C. pneumoniae.* The same effect was seen when active CPAF was expressed in non-infected cells and in a cell-free system. Ectopic expression of active CPAF in non-infected cells was sufficient for GA fragmentation. GA fragmentation required the small GTPases Rab6 and Rab11 downstream of CPAF-activity. These results define CPAF as the first protein that is essential for replication of *Chlamydia*. We suggest that this role makes CPAF a potential anti-infective therapeutic target.

## Author Summary

Chlamydiae are bacteria that replicate only inside host (for instance human) cells and that are frequent agents of human disease, in particular sexually transmitted disease. *Chlamydia* lives in a vacuole inside the cell, surrounded by a lipid membrane, and must acquire nutrients and other factors from the host cell for its replication and for the growth of the vacuole. Recent results show that for this, *Chlamydia* relies on its ability to induce the loss of an individual protein of the Golgi apparatus (a cellular structure that sorts materials for transport in the cell) called golgin-84. In this work we find that *Chlamydia* does this using its protein-cleaving enzyme CPAF (which is made by *Chlamydia* and transported from the vacuole into the cell). CPAF cleaves golgin-84 and thereby induces changes in the Golgi apparatus that are linked to the acquisition of some cellular material by *Chlamydia*. We further show that a synthetic inhibitor, which was recently found to block chlamydial growth, does that by inhibiting CPAF. CPAF therefore seems necessary for chlamydial growth and blocking CPAF may be a therapeutic strategy against infections with *Chlamydia*.

## Introduction

Chlamydiae are a group of obligate intracellular bacteria that infect humans and animals. *Chlamydia trachomatis* is the most common bacterial agent of sexually transmitted disease with a prevalence of up to about 5% in young women as well as a common cause of eye infections. Clinically, the most relevant aspect of infection with *C. trachomatis* is its propensity for chronic infection, which may lead to female infertility and to blinding trachoma [1], [2], [3]. *Chlamydia pneumoniae* is a very common cause of (typically mild) airway infection but it has also been proposed to cause chronic infection of artery walls, contributing to atherosclerosis [4].

During its developmental cycle *Chlamydia* switches between two morphologically distinguishable forms. The infectious but metabolically inactive elementary body (EB) is taken up by the host cell, where it resides in the cytosol, within a membranous vacuole, termed the inclusion. Within the inclusion EBs differentiate into reticulate bodies (RBs), which divide by binary fission. During this intracellular growth phase the inclusion substantially increases in size, often filling almost the entire cell at later time points. Towards the end of the cycle RBs re-differentiate into EBs, which are subsequently released from the cell. *In vitro* this cycle takes approximately 2 days for *C. trachomatis* and 3-4 days for *C. pneumoniae* [5].

To be able to grow, *Chlamydia* must escape cellular defenses and acquire nutrients and macromolecules from the host cell. In particular, trafficking of host lipids to the chlamydial inclusion has been noted where they are probably required for the expanding lipid membrane of the growing inclusion as well as for bacterial membranes [6], [7], [8]. Numerous alterations of other host cell systems have also been described during infection, including massive changes in gene transcription, cytoskeletal rearrangement and the inhibition of apoptosis [9]. Although molecular details often remain unclear these changes are probably mainly the result of the translocation of bacterial effectors into the host cytosol. *Chlamydia* possesses a type III secretion system and a number of bacterial proteins have been described to be secreted into the inclusion membrane or beyond [10], [11]. One chlamydial protease, chlamydial protease-like activity factor (CPAF), is known to translocate from the inclusion to the cytosol approximately mid-cycle [12]. Several host cell proteins have been identified as proteolytic targets of CPAF although the immediate relevance of such cleavage events for the development of the infection has not been established [13].

We recently identified the fragmentation of the Golgi apparatus (GA) as a consequence of infection with *C. trachomatis* [14], [15]. This fragmentation coincides with the cleavage of an integral GA matrix protein, golgin-84, and can be phenocopied by silencing of golgin-84 by RNAi. Intriguingly, golgin-84-cleavage can be inhibited by a modified tetrapeptide, z-WEHD-fmk (WEHD-fmk) [14], and WEHD-fmk blocks the replication of *C. trachomatis* [14], [16]. Conversely, silencing of golgin-84 enhances bacterial replication [14]. This strongly suggests that the cleavage of golgin-84 during infection causes fragmentation of the GA and that this fragmentation is required for chlamydial growth, most likely because it facilitates transport of essential lipids to the inclusion.

Here we identify CPAF as the chlamydial protease responsible for golgin-84-cleavage and as the target of WEHD-fmk during infection. WEHD-fmk, which has been developed as an inhibitor of caspase-1 is shown to inhibit CPAF-activity in a number of experimental situations. When expressed ectopically in human cells, CPAF is found to cause the fragmentation of the GA. *Chlamydia* thus depends on the secretion of CPAF into the host cell to ensure its intracellular growth. Targeting this individual bacterial protease can prevent replication of *Chlamydia* in human cells.

## Results

### WEHD-fmk inhibits growth and development of pathogenic *Chlamydia*

**Figure 1:**
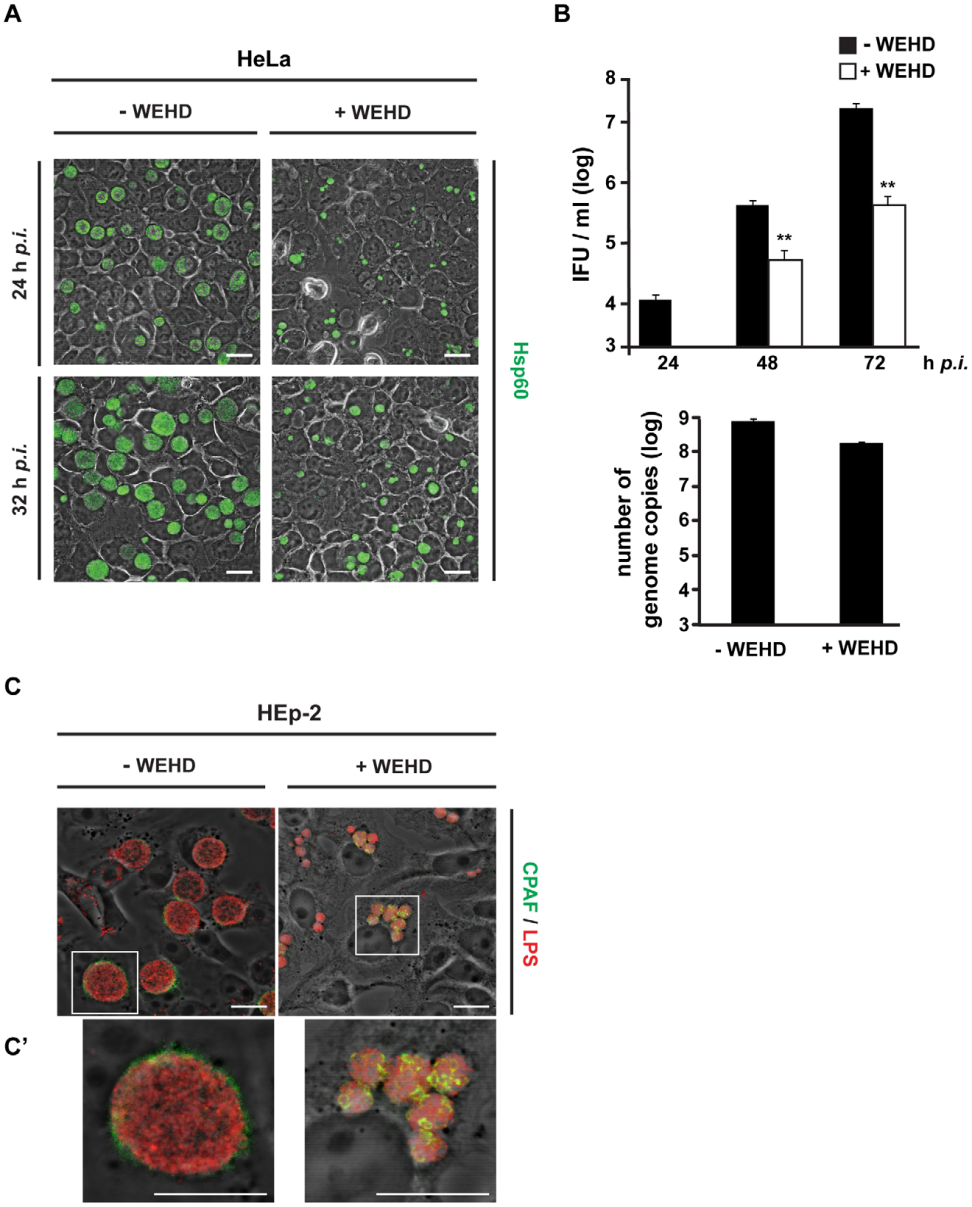
WEHD-fmk inhibits chlamydial growth. (A) WEHD-fmk inhibits the development of *C. trachomatis*. HeLa cells were infected with *C. trachomatis* (MOI = 2). At indicated time points cells were fixed, stained for bacterial Hsp60 (green) and analysed by confocal microscopy. Overlay with the phase contrast is shown. Scale bars, 10 µm. (B) WEHD-fmk reduces infectious progeny of *C. pneumoniae* in cell culture. Top: HEp-2 cells were infected with *C. pneumoniae* (MOI = 3). At 24 h *p.i*., WEHD-fmk or DMSO (solvent control) was added. Cell aliquots were harvested each day to a maximum of 96 h *p.i.* and infectious EBs were enumerated by titration on fresh host cell monolayers. Data are mean/SEM from three independent experiments. **, p-value < 0.05 (Students t-test). As WEHD-fmk was only added at 24 h, there is no value for cultures with the inhibitor at this point. Bottom: Genome copy number does not change after WEHD treatment. Hep2 cells were infected with *C. pneumoniae* using an MOI of 3. WEHD treatment (80 µM) was started 24h post infection. 2 days post infection, genome copy numbers were compared by quantitative real time PCR. (C) Localisation of *C. pneumoniae* CPAF in the presence of WEHD. HEp-2 cells were infected with *C. pneumoniae* (MOI = 3). 24 h *p.i.* cells were treated with 80 µM WEHD-fmk or DMSO. On day 3 cells were fixed and CPAF (green channel) and bacterial LPS (red channel) were visualized with specific antibodies. Samples were analysed by confocal laser scanning microscopy. Overlays of the two fluorescence channels and phase contrast are shown. Ć shows higher magnification of the boxed area. Scale bars, 10 µM.

We have previously shown that WEHD-fmk-treatment dramatically (several hundredfold) inhibits the generation of infectious EB during cell culture infection with *C. trachomatis* [14]. Confocal analysis confirmed that development of *C. trachomatis* was strongly reduced and inclusions were reduced in size when cultures were treated with WEHD-fmk (Fig. 1A).

We also determined that WEHD-fmk inhibits the growth of a chlamydial species distinct from *C. trachomatis*, *C. pneumoniae.* As shown in Fig. 1B, WEHD-fmk substantially inhibited growth and development of *C. pneumoniae* in infected epithelial cells. In *C. pneumoniae* infection WEHD-fmk impaired the production of infectious EBs more than 10-fold at 3 days post-infection (d *p.i.*) (Fig. 1B, left panel) although this effect was not quite as striking as in the case of *C. trachomatis* (see [14]). Genome copy number was measured at 48 h (following WEHD-fmk addition at 24 h) and was found to be about 4-fold reduced (Fig. 1B, bottom panel).

Inhibitor treatment was also demonstrated to impede the fusion of individual, smaller bacterial inclusions to larger inclusions during *C. pneumoniae* infection (Fig. 1C). During acute *C. pneumoniae* infection the chlamydial protease CPAF is secreted from the inclusion into the cytosol approximately mid-cycle [17]. We analysed CPAF localisation in *C. pneumoniae* infected cells 3 d *p.i*. by confocal microscopy and found that secretion of CPAF was blocked when cells were cultured in the presence of WEHD-fmk (Fig. 1C). An unusual, patchy staining pattern for CPAF was observed inside the inclusions in the presence of WEHD-fmk, whereas inhibitor treatment did not affect lipopolysaccharide (LPS) staining pattern. This suggests that less CPAF is secreted from the chlamydial inclusion into the host cell during *C. pneumonia* infection (Fig. 1C) (we used *C. pneumoniae* here because we were able to obtain better staining with the antibodies available to us in this species). As will become clear below, all of this probably reflects the general growth inhibition imposed by WEHD-fmk.

### WEHD-fmk blocks *Chlamydia*-dependent proteolytic activity

**Figure 2:**
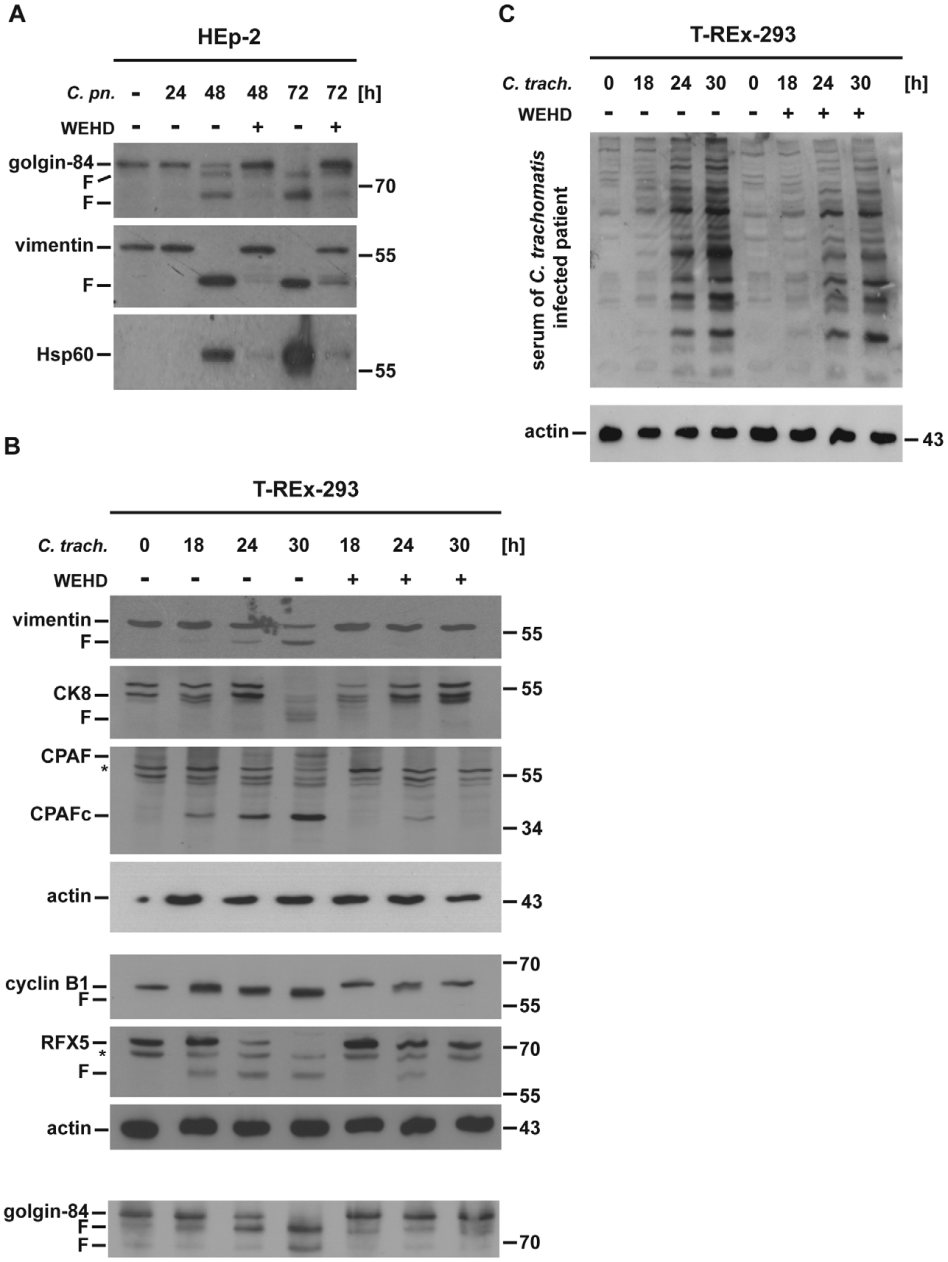
WEHD-fmk inhibits CPAF-dependent cleavage events during infection with *Chlamydia.* (A) Inhibition of proteolysis during infection with *C. pneumoniae*. HEp-2 cells were infected with *C. pn*eumoniae (MOI = 3). 24 h *p.i.* either 80 µM WEHD-FMK (+) or DMSO (-) were added. Cells were collected and lysed 24, 48 and 72 h *p.i.*. Uninfected cells were harvested after 24 h. Golgin-84, vimentin and bacterial Hsp60 were detected in lysates by immunoblotting. F, specific cleavage products. (B) Inhibition of proteolysis during infection with *C. trachomatis*. T-REx-293 cells were infected with *C. trachomatis* (MOI = 2) for the time points indicated. The peptide inhibitor WEHD-fmk (75 µM) was added after 9 h of infection. Cell extracts subjected to Western Blot analysis, probing for CPAF-substrates and CPAF. CPAF is expressed as an inactive precursor but is rapidly activated by autocatalytic cleavage. The antiserum was raised against a peptide in the C-terminal fragment of CPAF. Shown is a representative result of three independent experiments. *, non-specific background bands. F, CPAF specific cleavage products. CPAFc, C-terminal fragment of mature (proteolytically processed) CPAF. Three individual blots with samples from the same experiment are shown (vimentin, CK8, CPAF, actin; cyclin B1, RFX5, actin; golgin-84). (C) Assessment of chlamydial protein expression in the presence of WEHD-fmk. T-REx-293 cells were infected with *C. trachomatis* (MOI = 2) for the times indicated. The peptide inhibitor WEHD-fmk (75 µM) was added after 9 h of infection where stated. Cell extracts were subjected to Western Blot analysis, using a mix of sera from five patients that had tested positive for antibodies to *C. trachomatis* antibodies by routine clinical testing.

As previously reported for infection with various *C. trachomatis* strains [14], the infection with *C. pneumoniae* also caused the cleavage of the GA matrix protein, golgin-84 (Fig. 2A). However, the protease responsible for this degradation had not been identified. We considered the participation of the chlamydial protease CPAF mainly for two reasons. First, the time course of golgin-84-cleavage during infection (beginning around mid-cycle) correlates very well with the appearance of CPAF-activity [12], [17]. Indeed, CPAF-mediated degradation of vimentin correlated well with the degradation of golgin-84 during *C. pneumoniae* infection (Fig. 2A and see below). Secondly, we observed that WEHD-fmk not only inhibited the degradation of golgin-84 during *C. pneumoniae* infection but also prevented the cleavage of the known CPAF substrate vimentin (Fig. 2A).

To gain an overview of WEHD-fmk-specificity we tested a number of related tetrapeptide inhibitors. Screening of a library of inhibitors, which has been designed to inhibit various caspases, showed that among these modified peptides WEHD-fmk had the strongest CPAF-inhibitory effect in the cell-free assay (using recombinant CPAF) while two inhibitors, VEID-fmk and LEVD-fmk, came close in terms of inhibitory potential (Fig. S1A; we here used as a source of active CPAF 293T cells where CPAF had been activated by dimerisation; Text S1; [18] and see below). Others (for instance DEVD-fmk and LEHD-fmk; also YVAD-fmk, which has like WEHD-fmk been devised as a caspase-1-inhibitor) had no detectable activity. Closer investigation of some of the inhibitors showed that VEID-fmk was comparable to WEHD-fmk in the cell-free system (Fig. S1B; Text S1) and showed some, although less marked, activity in inhibiting CPAF-dependent cleavage inside human cells (Fig. S1C) or growth of *Chlamydia* (Fig. S1E, Text S1), most likely due to different cell-permeability.

We had previously found that a calpain inhibitor also blocked *Chlamydia*-mediated degradation of golgin-84 [14]. This inhibitor was also active in inhibiting CPAF-mediated vimentin cleavage in T-Rex-293-gyrB-CPAF cells (Fig. S1C). When tested during infection, LEHD-fmk and DEVD-fmk were inactive in inhibiting CPAF-mediated cleavage of vimentin or bacterial growth (Fig. S1D, E). This suggests that during chlamydial infection WEHD-fmk acts by targeting CPAF rather than a caspase.

For a closer analysis of the link between WEHD-fmk and CPAF we returned to the *C. trachomatis* cell culture infection model. Again, WEHD-fmk not only inhibited the degradation of golgin-84 but also the cleavage of the CPAF-substrates vimentin, cytokeratin-8 (CK8), cyclin B1 and RFX5 [13], [18] (Fig. 2B). Remarkably, WEHD-fmk also reduced the detectable levels of active CPAF (Fig. 2B). This is probably the consequence of a feedback-loop: since WEHD-fmk blocks the growth of *Chlamydia*, fewer bacteria develop in the cells and this reduces the total production of CPAF. Addition of CPAF also blocked the appearance of the strong anti-apoptotic effect that *Chlamydia* establishes in infected cells (i.e. in the presence of WEHD-fmk infected cells remained sensitive to staurosporine while they became resistant in its absence as reported before [19]; data not shown). In the presence of WEHD-fmk, there was a modest reduction in the amounts of chlamydial proteins, as assessed by probing Western blots with *Chlamydia*-reactive antisera from human patients (Fig. 2C), suggesting inhibition of growth or development. This inhibition very likely also leads to the defects in CPAF secretion and chlamydial development (see above).

### WEHD-fmk targets CPAF

These data were suggestive of a link between growth inhibition of *Chlamydia* by WEHD-fmk and CPAF. Possible mechanisms of the observed effects included the reduced secretion of CPAF from the vacuole to the cytosol and the direct inhibition of CPAF by WEHD-fmk. It could also not be excluded at this stage that the loss of CPAF activity was secondary to a different inhibitory mechanism of WEHD-fmk.

To distinguish between these possibilities we used a system for the ectopic expression of active CPAF in human cells. As reported earlier, CPAF can be activated in the absence of infection by the experimental dimerisation of CPAF (through the dimerisation of its fusion partner gyrase B (gyrB), which is achieved by the dimeric gyrB ligand coumermycin (CM) [18]). We utilised a system based on the human cell line 293T, which stably carries a tetracycline-inducible gyrB-*C. trachomatis*-CPAF construct (the cells are designated T-REx-293-gyrB-CPAF). Addition of anhydrotetracycline (AHT, a tetracycline-derivative lacking antibiotic activity) and CM to these cells induces CPAF-activation as measured as autocatalytic cleavage and cleavage of all known CPAF-substrates [18].

**Figure 3:**
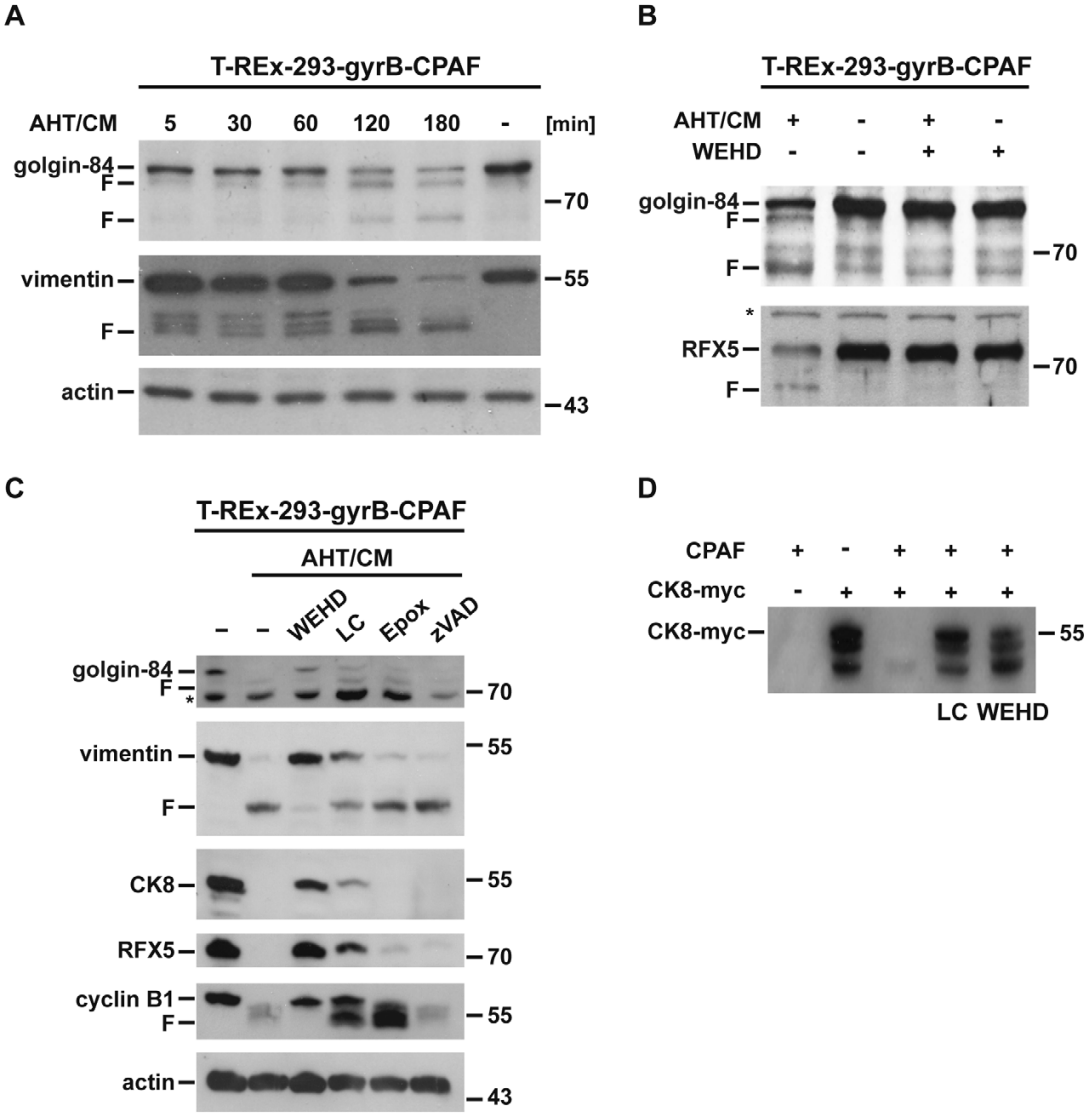
WEHD-fmk is an inhibitor of CPAF-dependent proteolysis. (A) CPAF cleaves golgin-84. Active CPAF was expressed in T-REx-293-gyrB-CPAF cells. For this, cells were stimulated with AHT for 16 h to express CPAF-precursor. The CM was added to activate CPAF. At the time points indicated after CM addition, cells were harvested, lysed and golgin-84 and vimentin were detected by immunoblotting. F, CPAF specific cleavage products. (B) WEHD-fmk blocks degradation of golgin-84 and RFX5 in T-REx-293-gyrB-CPAF cells. Cells were either treated simultaneously with AHT/CM for 12 h to activate CPAF or were left untreated. CPAF-activity was inhibited by 80 µM WEHD-fmk or DMSO was added. Cell extracts were prepared and probed for the proteins indicated. *, non-specific background band. F, CPAF specific cleavage products. (C) Inhibition profile of golgin-84 degradation. T-REx-293-gyrB-CPAF cells were treated with WEHD-fmk (75 µM), lactacystin (25 µM), the proteasome inhibitor epoxomicin (2.5 µM) or the pan-caspase inhibitor z-VAD-fmk (75 µM) prior to CPAF-induction and activation (by AHT/CM for 18 h). Cell extracts were prepared and subjected to Western Blot analysis. Shown is a representative result of four independent experiments. F, CPAF specific cleavage products. (D) Inhibition of CPAF-activity by WEHD-fmk in a cell-free system. Cell extracts of T-REx-293-gyrB-CPAF cells and T-REx-293 cells expressing myc-tagged cytokeratin 8 (CK8-myc) were combined in the presence of either WEHD-fmk (75 µM) or lactacystin (40 µM) and incubated for 60 min at 37°C. Reactions were analyzed by Western blotting. A-C: Shown is a representative result of three independent experiments. F, proteolytic product. LC, lactacystin. Epox, epoxomicin. zVAD, zVAD-fmk.

Expression of active CPAF in T-REx-293-gyrB-CPAF cells caused the cleavage of vimentin as reported earlier (Fig. 3A) [18]. With a similar time course golgin-84 was degraded to fragments that were indistinguishable from those generated during chlamydial infection (Fig. 3A) suggesting that CPAF was responsible for the cleavage of golgin-84 in both situations. Addition of WEHD-fmk at the time of CPAF-induction prevented the cleavage of golgin-84 as well as the degradation of the known CPAF-substrates (Fig. 3B, C). Lactacystin, which is known to inhibit both the human proteasome and CPAF [12], [20], also reduced cleavage of the CPAF-substrates tested while a specific inhibitor of the proteasome (epoxomicin) had no effect (Fig. 3C).

WEHD-fmk has been developed as an inhibitor of caspase-1. Since caspase-1-activation during infection of HeLa cells has recently been demonstrated in *Chlamydia* infected cells [16], it was also a possibility that part of the effect of WEHD-fmk was due to caspase-1 inhibition. We have above shown evidence that WEHD-fmk is not acting by inhibiting a caspase, and the evidence is overwhelming that the CPAF-dependent cleavage of the host cell proteins tested (vimentin, CK8, RFX5, cyclin B1) does not depend on caspase-1 [12], [13], [18], [20], [21], [22]. The pan-caspase-inhibitor z-VAD-fmk further failed to inhibit the tested cleavage events (Fig. 3C), excluding the possibility that WEHD-fmk acted by inhibition of caspases in this system.

These results suggested that WEHD-fmk acted as an inhibitor of CPAF. This notion is further supported by the finding that WEHD-fmk also blocked the morphological effects that CPAF produces when expressed in T-REx-293-gyrB-CPAF cells (Fig.S2A; Text S1) at concentrations that blocked cleavage of vimentin (Fig. S2B). In a cell-free assay, lysate from AHT/CM-induced T-REx-293-gyrB-CPAF cells (i.e. lysate containing active CPAF) degraded myc-tagged CK8 (separately expressed by transfection of 293T cells); this cleavage was inhibited by WEHD-fmk and lactacystin (Fig. 3D). Further, bacterial lysate containing active recombinant CPAF degraded the substrates vimentin and CK8 in lysates from human cells and this cleavage was again inhibited by WEHD-fmk (Fig. S3A, B; Text S1). Thus, cleavage of all tested proteins that are known to be cleaved in a CPAF-dependent way during infection was inhibited by WEHD-fmk. Further, the cleavage of the same proteins was also blocked by WEHD-fmk when active CPAF was expressed in human cells, as was the cleavage of vimentin and CK8 by bacterial recombinant CPAF. This makes it very unlikely that another protease was involved and strongly suggests that WEHD-fmk acted as an inhibitor of chlamydial CPAF.

We have shown previously that most of the inhibitory effect of WEHD-fmk is reversed when golgin-84 is knocked down, suggesting that the effect of the inhibitor is limited to preventing the cleavage of golgin-84 (and therefore to inhibiting CPAF [14]). Inhibition of chlamydial replication by WEHD-fmk is therefore very likely due to CPAF inhibition and not effects on additional targets.

### Ectopically expressed active CPAF causes GA fragmentation, dependent on Rab6A and Rab11A

**Figure 4:**
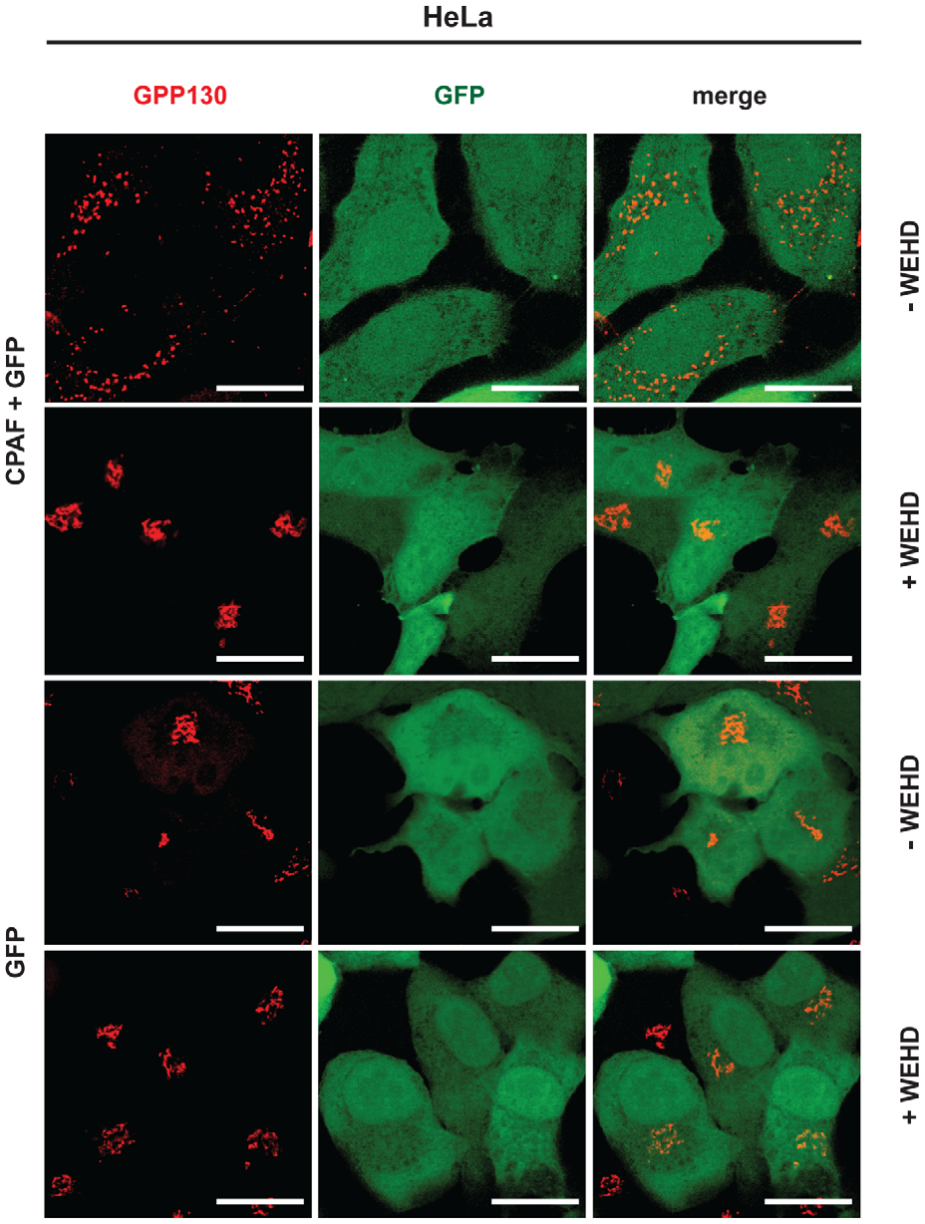
CPAF causes Golgi-complex fragmentation. Transiently expressed CPAF causes Golgi fragmentation in epithelial cells. HeLa cells were either cotransfected with constructs coding for gyrB-CPAF and pCMV-eGFP (CPAF + GFP) or transfected with the GFP-construct only (GFP). CPAF was induced and activated 4h post transfection by adding AHT/CM and WEHD (75 µM) was added at the same time (+WEHD). Fragmentation of the Golgi apparatus was analyzed 48 h post induction by staining of the GA matrix protein GPP130. *Left*: GPP130 staining; *middle*: GFP expression, *right*: merged images. Scale bars, 20 µM.

As reported previously, *Chlamydia* causes the fragmentation of the GA to ensure its replication, very likely because this facilitates lipid transport to the bacterial inclusion [14], [15]. Since GA fragmentation is linked to the cleavage of golgin-84 and golgin-84-cleavage is the result of CPAF-activity, CPAF-activity may be expected to be responsible for the fragmentation of the GA during infection. Indeed, the isolated expression of active CPAF induced GA fragmentation in T-REx-293-gyrB-CPAF cells (Fig. S4; Text S1) as well as in HeLa cells transiently expressing the gyrB-CPAF expression construct (Fig.4). The presence of WEHD-fmk during expression of active CPAF prevented GA fragmentation as expected (Fig. 4, Fig. S4).

**Figure 5:**
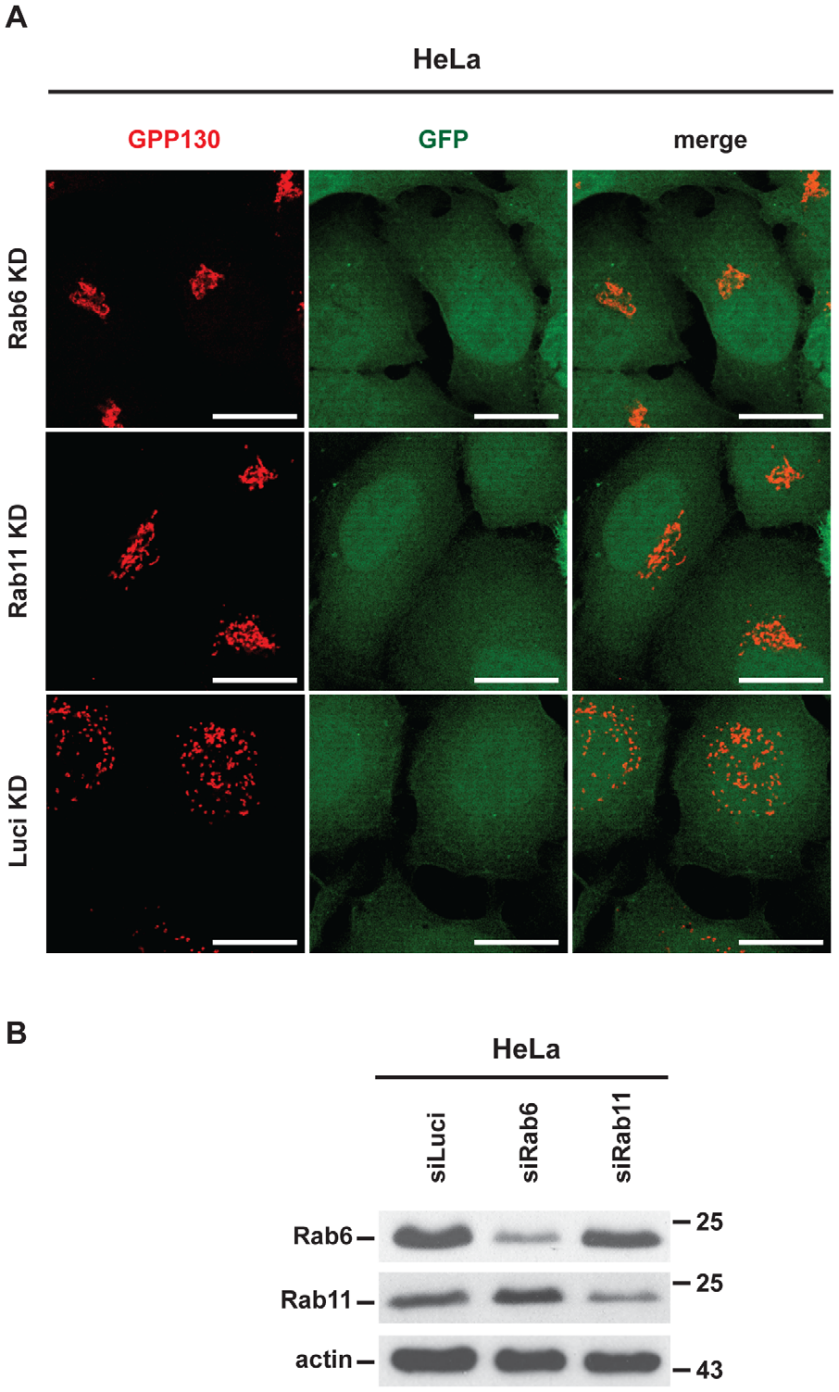
CPAF-induced Golgi fragmentation is Rab6A- and Rab11A-dependent in HeLa cells transiently expressing active CPAF. (A) HeLa cells were depleted for Rab6A, Rab11A by transfection with specific siRNAs. Luciferase-specific siRNA was used as a control. 48 h post transfection of the siRNAs, an expression construct for gyrB-CPAF was cotransfected with pCMV-eGFP. CPAF was induced and activated 4h post transfection by adding AHT/CM. 48 h post transfection of CPAF- and GFP-vectors, cells were fixed and immunostained. *Left*: GPP130, *middle*: GFP expression, *right*: merged images. Scale bar, 20 µm. (B) Expression of Rab6A and Rab11A after siRNA transfection. HeLa cells were transfected with siRNAs specific for Rab6A, Rab11A or luciferase. Protein expression was assessed after 72 h by Western blotting.

We have recently shown that *Chlamydia*-induced GA fragmentation requires the small GTPases Rab6A and Rab11A [15]. If CPAF is responsible for GA fragmentation, this effect of CPAF is expected also to depend on Rab6 and Rab11. We tested this prediction and found that indeed the reduction of either Rab6 or Rab11 by RNAi had a clear inhibitory effect on GA fragmentation induced by the expression of active CPAF in HeLa cells (Fig. 5) while knock-down per se did not affect GA morphology (Fig. S5; Text S1) [15].

Taken together, these results provide strong evidence that CPAF is the enzyme that cleaves golgin-84 and thereby contributes to GA fragmentation, which in turn is linked to lipid transport to the chlamydial inclusion. WEHD-fmk blocks CPAF activity, interrupting these events and inhibiting normal chlamydial growth.

## Discussion

These results establish a link between the production of CPAF by *Chlamydia*, the cleavage of golgin-84 and subsequent GA fragmentation, which again is required for efficient chlamydial replication. CPAF activity is thus indispensable for the intracellular replication and development of *Chlamydia*, and the targeting of CPAF may be a strategy to reduce the burden of chlamydial infection.

WEHD-fmk blocked the degradation of all known and investigated CPAF substrates when recombinant CPAF was tested in cell extracts and when active CPAF was expressed in human cells. This suggests a direct binding of the inhibitor to the protease. Although the protease has been crystallised together with its known inhibitor lactacystin, the great width of the active site makes it difficult to predict how this inhibitory mechanism might work. The differences in inhibitory potential of other peptide inhibitors tested suggest a binding of individual amino acids from the inhibitor sequence. There is no clearly defined substrate recognition and cleavage sequence of CPAF. The possibility should also be considered that substrate recognition of CPAF occurs at a site distinct from the actual cleavage site. If that was the case, WEHD-fmk might bind at a domain outside the active site of the protease.

During infection, WEHD-fmk not only inhibits CPAF-dependent substrate cleavage but also growth of *Chlamydia*, as already reported earlier [14]. This is linked to the cleavage of golgin-84 and the subsequent GA fragmentation. CPAF has a high number of known substrates [13] and very likely many more that have not been identified. All of these substrates may have an effect on chlamydial growth. However, the cleavage of golgin-84 may be of particular importance. As we have found earlier, knock-down of golgin-84 (inducing GA fragmentation) reverses the inhibitory effect of WEHD-fmk to a considerable extent [14]. As cleavage of all other CPAF substrates is presumably still blocked by the inhibitor in this situation, these cleavage events may not be critical, at least in cell culture.

The group of chlamydiae and *Chlamydia*-related bacteria are very widespread and successful as infectious agents of many diverse species throughout the animal kingdom [23]. The forms of related bacteria range from organisms that have been isolated as symbionts of free-living amoebae (for instance *Protochlamydia amoebophila* [24]) over agents that parasitize amoebae but may also infect humans (*Parachlamydia acanthamoebae* [25]) to a number of species that frequently infect animal (such as *C. caviae, C. abortus*) and human hosts (*C. trachomatis, C. pneumoniae, C. psittaci*). All of these bacterial species share the lifestyle of intracellular development in a membranous cytosolic inclusion. This developmental peculiarity carries the advantage of a degree of protection against host cell defense systems afforded by the lipid membrane, and probably makes it easier to generate a specialised environment for optimal replication. It is however also associated with the requirement of arranging for vacuole growth by continuous substitution of lipids as well as the acquisition of lipids and nutrients from the host cell for bacterial growth.

Sphinogmyelin acquisition by the chlamydial inclusion occurs both early (within hours of infection) and late in the developmental cycle, and has been linked to multivesicular bodies and to interception of post-Golgi traffic [7], [8], [26]. We have recently found that fragmentation of the GA facilitates this transport at later stages of infection [14]. The results shown here suggest that this late sphingolipid acquisition is the result of CPAF activity, which may be an important aspect of the function of this protease in chlamydial intracellular development.

Identifiable CPAF-orthologs have been found in *Chlamydia*-related organisms as distant from human pathogens as *P. amoebophila* [24]. This suggests that CPAF indeed serves a function that is indispensable to the lifestyle of these organisms. It is at least conceivable (although highly speculative at this stage) that the function in vesicle acquisition that we identify here during infection of human cells with *C. trachomatis* was an original function of CPAF already in *Chlamydia*-related organisms parasitizing amoebae.

Acute chlamydial infections can be easily and successfully treated with antibiotics and although antibiotic resistance may develop this is not a major problem at this stage. The biggest problem clinically concerns chronic infections. At least *in vitro*, chlamydiae can enter a persistent state that likely reflects chronic infection and during which *Chlamydia* is resilient to antibiotic treatment [27]. We cannot say whether *Chlamydia* also depends on CPAF for the maintenance of a chronic infection but it is intriguing that serum antibodies to CPAF are characteristic of long-term infections in humans [28], suggesting that CPAF is indeed expressed during chronic infection. The inhibition of CPAF, the principal feasibility of which is shown here, may therefore turn out to be a valuable approach to the therapy of human chlamydial infection.

## Materials and Methods

### Cell lines and culture

The human embryonic kidney cell line T-REx-293, which stably expresses the tetracycline repressor (Invitrogen, Darmstadt, Germany) was maintained in humidified air at 5% CO_2_ and 37°C in Dulbecco modified Eaglès minimal essential medium (DMEM) supplemented with 10% fetal calf serum (FCS, tetracycline negative; PAA Laboratories, Pasching, Austria) and 5 µg/ml blasticidin (PAA Laboratories, Pasching, Austria). Stable, gyrB-CPAF expressing T-REx-293 clones were generated by electroporation with the pcDNA4/TO/*myc*-His-gyrB-CPAF (*C. trachomatis* CPAF) construct and subjected to antibiotic selection as previously reported [18]. The cells were cultured as above but in the presence of zeocin (350 µg/ml; InvivoGen, San Diego, CA, USA). To induce and activate CPAF 5 ng/µl anhydrotetracycline (AHT; IBA, Göttingen, Germany) and 1 µM coumermycin (CM; Sigma-Aldrich, Steinbach, Germany) were added. HeLa and HEp-2 cells were grown in DMEM supplemented with 10% fetal calf serum but lacking blasticidin and zeocin. WEHD-fmk (R&D Systems, Wiesbaden-Nordenstedt, Germany), z-VAD-fmk (Bachem, Bubendorf, Switzerland), epoxomicin (Calbiochem, Darmstadt, Germany) or Clasto-lactacystin β-lactone (Sigma-Aldrich, Steinbach, Germany) were added as indicated.

### Chlamydial infection

The *C. trachomatis* strain (serovar L2) was obtained from the American Type Culture Collection (ATCC). For infection of T-REx-293 or HeLa cells, the culture medium was replaced with serum free medium without antibiotics and bacteria were added using the specified multiplicity of infection (MOI). Two hours later the medium was supplemented with 10% FCS. *C. pneumoniae* strain VR1310 was a kind gift of Dr. Gunnar Christiansen (Institute of Medical Microbiology and Immunology, University of Aarhus, Denmark). Prior to infection, culture medium was removed and *C. pneumoniae* was added in DMEM containing 1 µg/ml cycloheximide. The cells were then centrifuged at 2,000 *g* for 35 min at 35°C and further cultivated for the times indicated.

### Immunoblotting

Cell extracts were prepared using RIPA-buffer (1% Triton X-100, 0.5% SDS, 0.5% deoxycholate, 1 mM EDTA, 150 mM NaCl, and 50 mM Tris, pH 8.0) supplemented with a protease inhibitor cocktail (Roche, Mannheim, Germany). Proteins were separated by SDS-PAGE and transferred onto nitrocellulose membranes. Antibodies used were directed against β-actin (Sigma-Aldrich, Steinbach, Germany), chlamydial Hsp60 (Alexis Biochemicals, San Diego, CA, USA), Bim, cyclin B1, myc (all from Cell Signaling Technology, Beverly, MA, USA), CK8, RFX5, vimentin (all from Acris, Herford, Germany), golgin-84 (BD Bioscience, Heidelberg, Germany) and CPAF (a polyclonal serum generated by immunization of rabbits with a peptide from the C-terminal fragment of *C. trachomatis* CPAF; Pineda Antibody-Service, Berlin, Germany). Antibodies specific for *C. pneumoniae* CPAF were a generous gift of Dr. Guangming Zhong (San Antonio, Texas, USA). In one series of experiments, Western blots were probed with human sera from patients that had tested positive for *C. trachomatis*-specific antibodies by line blot (Mikrogen, Munich, Germany). A mixture of five sera was used. Proteins were visualized using peroxidase-conjugated secondary antibodies and a chemoluminescence detection system (GE Healthcare, Uppsala, Sweden).

### Cell-free proteolysis assay

As a source of active CPAF, T-REx-293-gyrB-CPAF cells were stimulated with AHT/CM for 18 h and lysed in NP-40 buffer (1% NP-40, 150 mM NaCl, 1 mM EDTA, and 20 mM MOPS, pH 7.4). Cell extracts were mixed with equal volumes of lysate from T-REx-293 cells transiently transfected with a myc-tagged CK8-construct. Reactions were incubated at 37°C for 1 h and subjected to Western blot analysis. Clasto-lactacystin β-lactone or WEHD-fmk was added 30 min prior to substrate addition.

### RNA interference

siRNAs were purchased from QIAGEN (QIAGEN, Hilden, Germany). HeLa cells were seeded into 12-well plates one day before transfection. Transfection was performed using QIAGEN RNAiFect according to the manufacturer's guidelines. 1 µg siRNA was added to 96 µl Opti-MEM medium (Invitrogen, Darmstadt, Germany), vortexed, mixed with 6 µl RNAiFect and incubated for 15 min at room temperature. The liposome/RNA mix was added to cells with 600 µl growth medium [15]. After 24 h, cells were trypsinised and seeded into new cell culture plates and incubated for another 24 h.

### Immunofluorescence and confocal microscopy

Cells were seeded onto coverslips and treated as indicated. Cells were then fixed with 2% PFA for 30 min at room temperature. The Golgi marker GPP130 (Covance, Princeton, NJ, USA) as well as CPAF were detected using specific antibodies. Bacteria were stained using either a chlamydial LPS specific antibody (Milan Analytica AG) or a chlamydial Hsp60 specific antibody (Alexis Biochemicals, San Diego, CA, USA). Binding was visualized using fluorescence labeled secondary antibodies. Cells were mounted in Mowiol. For the localisation of CPAF, HeLa cells were infected with *C. pneumoniae* VR1310 (MOI = 3) and WEHD-fmk (80 µM) was added 24 h p.i. (DMSO was added in the controls). Fixation was followed by treatment with 0.2% Triton X-100 in PBS supplemented with 0.2% BSA. Cells were analysed with an LSCM (Leica TCS SP-1, 63x/1.32 HCX PL APO CS oil lens, Leica Microsystems, Wetzlar, Germany) and images were processed using Adobe Photoshop.

### Determination of *C. pneumoniae* genome copy numbers

Genome copy numbers were determined by real-time LightCycler PCR as described previously [29]. Briefly, DNA extraction was performed using NucleoSpin RNA/DNA according to the manufacturer's recommendations (Macherey-Nagel, Düren, Germany): The 16S rRNA was amplified (forward primer, CAT CGT TTA CGG CAA GGA CTA; reverse primer, AGG CCT TAG GGT TGT AAA GCA). Absolute numbers of genome copies were obtained by calculating the *cp-values* of the respective samples against a standard curve (ten-fold dilutions of *C. pneumoniae* infection inoculums) with known IFUs/ml.

## Supporting Information

Figure S1 **Peptide inhibitors vary in their efficacy in inhibiting CPAF and chlamydial growth.** (A) CPAF-inhibitory activity of inhibitors in a cell-free system. Cell extracts of T-REx-293-gyrB-CPAF cells and T-REx-293 cells expressing myc-tagged cytokeratin 8 (CK8-myc) were combined in the presence of lactacystin (LC, 40 µM), WEHD-fmk (75 µM) or peptide-fmk inhibitors of a Caspase-Family Inhibitor Set (Promokine, Heidelberg, Germany; 75 µM) and incubated for 60 min at 37°C. Reactions were analyzed by Western blotting. Shown is a representative result of two independent experiments. (B) Activity of recombinant CPAF is blocked by WEHD-fmk and VEID-fmk. Purified, recombinant CPAF was combined with lysate of T-REx-293 cells. Prior to the addition of CPAF substrate, WEHD-fmk or VEID-fmk was added to CPAF for 30 min as indicated. Reactions were incubated for 60 min at 37°C and analyzed by Western blotting. Shown is a representative result of three independent experiments. F, CPAF specific cleavage products. (C) VEID-fmk and calpain inhibitor III block degradation of vimentin in T-REx-293-gyrB-CPAF cells. Cells were treated with AHT/CM as indicated either alone or in the presence of VEID-fmk (75 µM), WEHD-fmk (75 µM) or calpain inhibitor III (100 µM; Calbiochem, Darmstadt, Germany). Cell extracts were prepared and probed for vimentin. In all cases representative results of two independent experiments are shown. In the bottom blot one lane was removed digitally. F, CPAF specific cleavage products. (D) WEHD- or VEID- but not LEHD- or DEVD-fmk inhibits CPAF-dependent cleavage of vimentin during chlamydial infection. T-REx-293 cells were infected with *C. trachomatis* (MOI = 2) for 24 h. WEHD-fmk, VEID-fmk, LEHD-fmk or DEVD-fmk (75 µM) were added after 9 h of infection. Cell extracts were subjected to Western blot analysis. Similar results were seen in three experiments. In two further experiments, the effect of VEID-fmk was smaller than that of WEHD-fmk. F, CPAF specific cleavage products. Intensities of bands corresponding to full length or cleaved protein were determined using the ImageJ software (http://rsbweb.nih.gov/). Ratios of fragment to intact protein in the various lanes are given below the blot. (E) WEHD- or VEID- but not LEHD- or DEVD-fmk inhibits chlamydial growth. HeLa cells were infected with *C. trachomatis* (MOI = 2). Inhibitors (75 µM) were added 8 h after infection. After 24 h cells were fixed, stained for chlamydial MOMP and analyzed by confocal microscopy. Overlay with the phase contrast is shown. Scale bar, 20 µm.
(TIF)

Figure S2 **WEHD-fmk blocks the consequences of CPAF-activity in CPAF expressing T-REx-293-gyrB-CPAF cells.** (A) WEHD-fmk suppresses CPAF-induced changes in cell morphology. Expression and activation of CPAF was induced in T-REx-293-gyrB-CPAF cells by addition of 5 ng/ml AHT and 1 mM CM for 18 h. WEHD-fmk, the pan-caspase inhibitor zVAD-fmk (75 µM). Inhibitors were added 30 min prior to CPAF-induction. Shown is a representative result of three independent experiments. Scale bar, 50 µm. (B) WEHD-fmk prevents CPAF-dependent vimentin-cleavage. T-REx-293-gyrB-CPAF cells were treated as in (A), lysed and subjected to Western blot analysis. Shown is a representative result of three independent experiments. F, CPAF specific cleavage product.
(TIF)

Figure S3 **CPAF-containing bacterial lysates exhibit cleavage activity and WEHD sensitivity.** (A) WEHD-fmk inhibits cleavage activity of CPAF-containing bacterial lysate. As above CPAF-containing *E. coli* extract was combined with lysates of transiently transfected T-REx-293 cells (vim-GFP or CK8-myc). Prior to the addition of CPAF substrate WEHD-fmk (100 µM) was added for 30 min. Lysates were incubated for 30 min at 37°C and analysed by Western blotting. Shown is a representative result of three independent experiments. *, non-specific background band. F, CPAF specific cleavage products. (B) CPAF-containing bacterial lysate cleaves CPAF substrates. CPAF-containing *E. coli* extract was combined with lysates of either vimentin-GFP (vim-GFP) or CK8-myc expressing T-REx-293 cells and incubated for the time points indicated at 37°C. Substrate cleavage was monitored by Western blotting. Untransformed bacteria or *E. coli* transformed with empty vector were used as controls. Shown is a representative result of three independent experiments. *, non-specific background band. F, CPAF specific cleavage products.
(TIF)

Figure S4 **WEHD-fmk inhibits Golgi fragmentation in CPAF expressing cells.** CPAF was induced and activated in T-REx-293-gyrB-CPAF cells using AHT/CM (5 h). Another set of cells were left untreated. WEHD-fmk (80 µM) was added at the time of induction (+ WEHD). Cells were fixed and Golgi fragmentation was analysed by GPP130 staining (red channel). Cells were counterstained using the DRAQ 5 (blue channel). Left: GPP130; center: DRAQ 5; right: merged images. Scale bar, 20 µm.
(TIF)

Figure S5 **Depletion of Rab6A or Rab11A does not alter GA morphology.** HeLa cells were transfected with siRNA specific for Rab6A, Rab11A or Luciferase. 48 h after siRNA transfection, gyrB-CPAF and CMV-eGFP were cotransfected. Cells were fixed and immunostained after an incubation of 48 h. Left: GPP130; middle: GFP; right: merged images. Scale bar, 20 µM.
(TIF)

Text S1 **Supporting Information: Experimental Procedures.**
(DOC)
