# Supplementary figures and images for: Targeting of a Chlamydial Protease Impedes Intracellular Bacterial Growth
Source: PLoS Pathog. 2011 Sep 29;7(9):e1002283. doi: 10.1371/journal.ppat.1002283 (PMC3182938; doi:10.1371/journal.ppat.1002283)

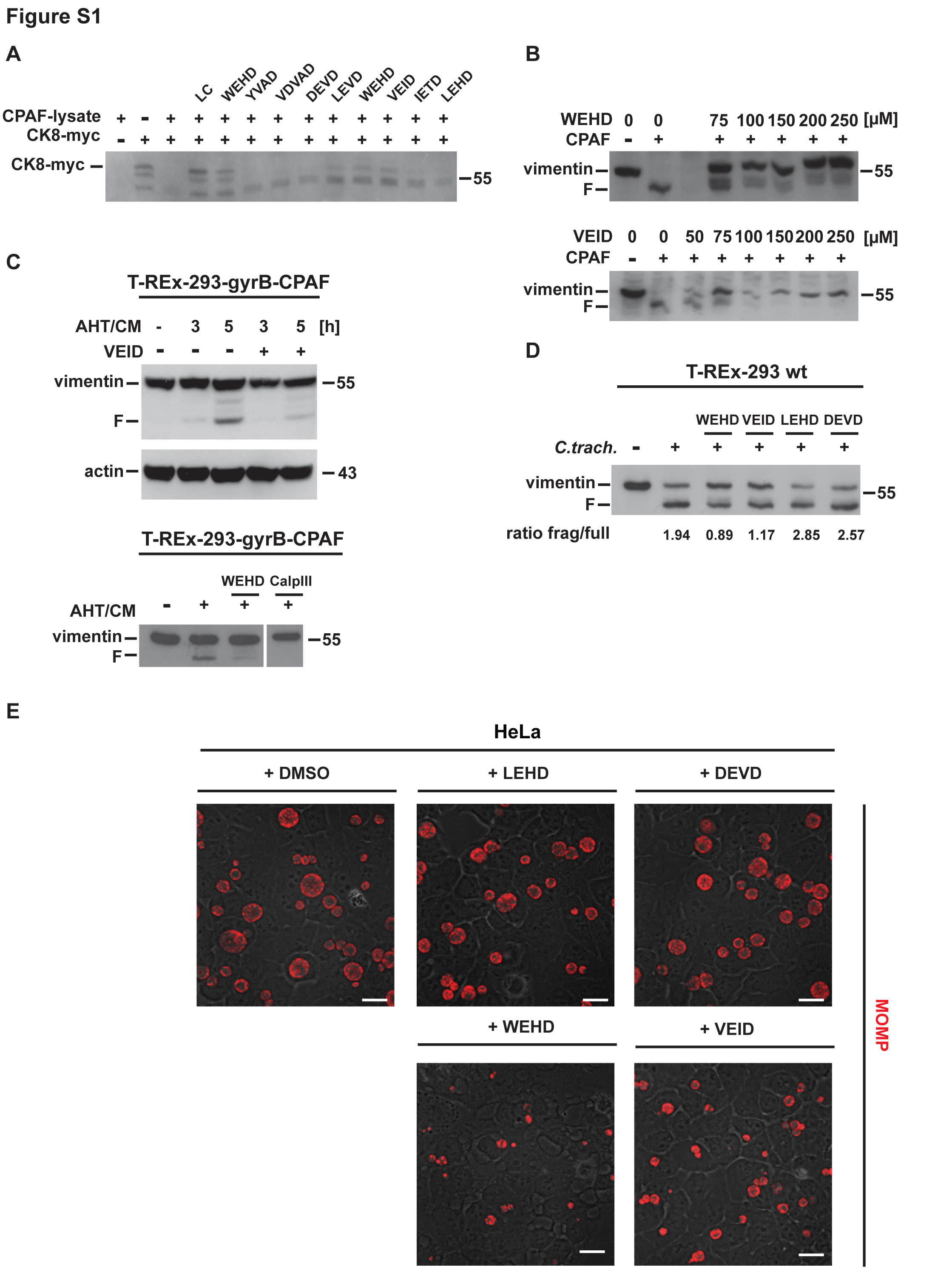

Supplement: Figure S1 — Peptide inhibitors vary in their efficacy in inhibiting CPAF and chlamydial growth. (A) CPAF-inhibitory activity of inhibitors in a cell-free system. Cell extracts of T-REx-293-gyrB-CPAF cells and T-REx-293 cells expressing myc-tagged cytokeratin 8 (CK8-myc) were combined in the presence of lactacystin (LC, 40 µM), WEHD-fmk (75 µM) or peptide-fmk inhibitors of a Caspase-Family Inhibitor Set (Promokine, Heidelberg, Germany; 75 µM) and incubated for 60 min at 37°C. Reactions were analyzed by Western blotting. Shown is a representative result of two independent experiments. (B) Activity of recombinant CPAF is blocked by WEHD-fmk and VEID-fmk. Purified, recombinant CPAF was combined with lysate of T-REx-293 cells. Prior to the addition of CPAF substrate, WEHD-fmk or VEID-fmk was added to CPAF for 30 min as indicated. Reactions were incubated for 60 min at 37°C and analyzed by Western blotting. Shown is a representative result of three independent experiments. F, CPAF specific cleavage products. (C) VEID-fmk and calpain inhibitor III block degradation of vimentin in T-REx-293-gyrB-CPAF cells. Cells were treated with AHT/CM as indicated either alone or in the presence of VEID-fmk (75 µM), WEHD-fmk (75 µM) or calpain inhibitor III (100 µM; Calbiochem, Darmstadt, Germany). Cell extracts were prepared and probed for vimentin. In all cases representative results of two independent experiments are shown. In the bottom blot one lane was removed digitally. F, CPAF specific cleavage products. (D) WEHD- or VEID- but not LEHD- or DEVD-fmk inhibits CPAF-dependent cleavage of vimentin during chlamydial infection. T-REx-293 cells were infected with C. trachomatis (MOI = 2) for 24 h. WEHD-fmk, VEID-fmk, LEHD-fmk or DEVD-fmk (75 µM) were added after 9 h of infection. Cell extracts were subjected to Western blot analysis. Similar results were seen in three experiments. In two further experiments, the effect of VEID-fmk was smaller than that of WEHD-fmk. F, CPAF specific cleavage products [file ppat.1002283.s001.tif]

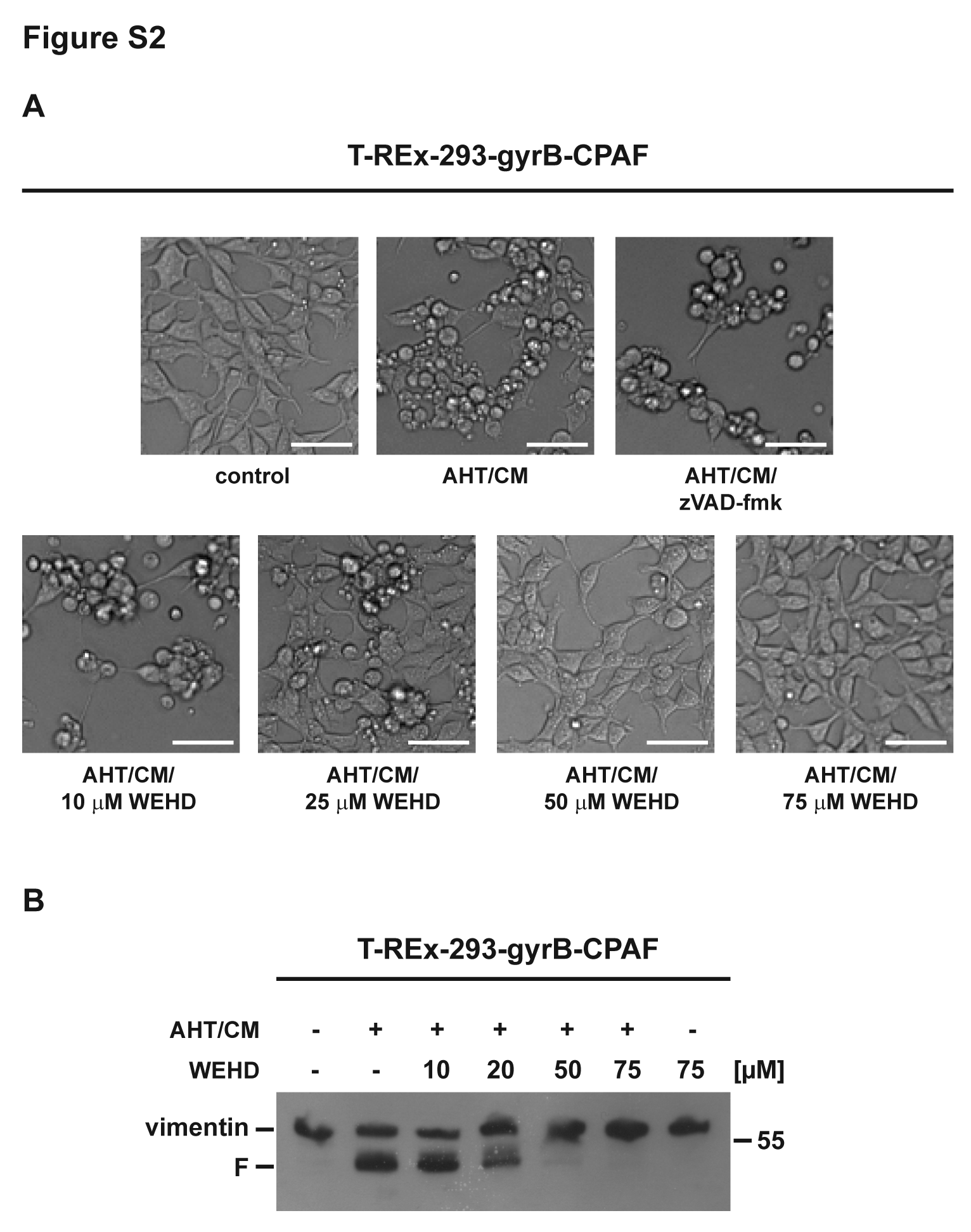

Supplement: Figure S2 — WEHD-fmk blocks the consequences of CPAF-activity in CPAF expressing T-REx-293-gyrB-CPAF cells. (A) WEHD-fmk suppresses CPAF-induced changes in cell morphology. Expression and activation of CPAF was induced in T-REx-293-gyrB-CPAF cells by addition of 5 ng/ml AHT and 1 mM CM for 18 h. WEHD-fmk, the pan-caspase inhibitor zVAD-fmk (75 µM). Inhibitors were added 30 min prior to CPAF-induction. Shown is a representative result of three independent experiments. Scale bar, 50 µm. (B) WEHD-fmk prevents CPAF-dependent vimentin-cleavage. T-REx-293-gyrB-CPAF cells were treated as in (A), lysed and subjected to Western blot analysis. Shown is a representative result of three independent experiments. F, CPAF specific cleavage product. (TIF) [file ppat.1002283.s002.tif]

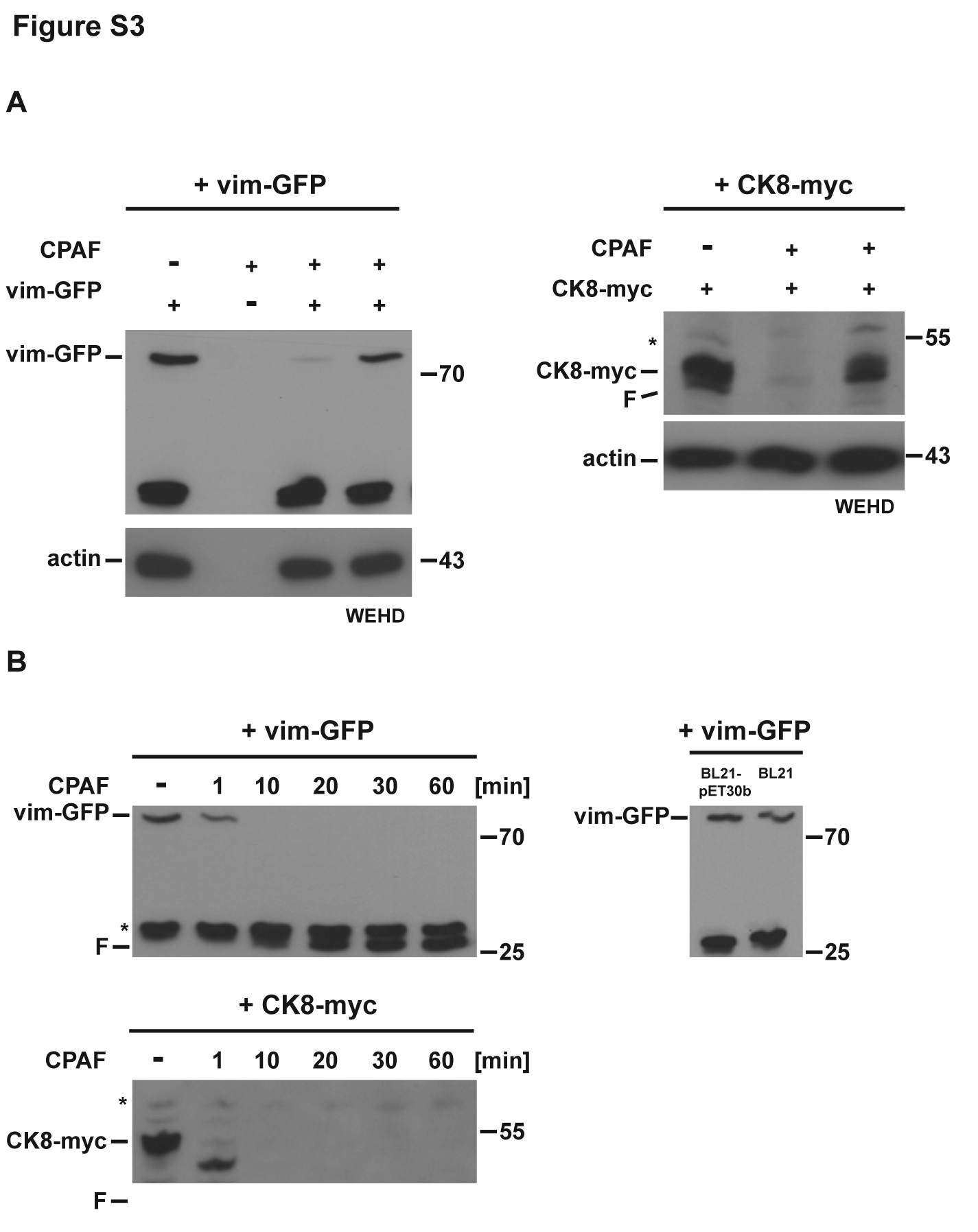

Supplement: Figure S3 — CPAF-containing bacterial lysates exhibit cleavage activity and WEHD sensitivity. (A) WEHD-fmk inhibits cleavage activity of CPAF-containing bacterial lysate. As above CPAF-containing E. coli extract was combined with lysates of transiently transfected T-REx-293 cells (vim-GFP or CK8-myc). Prior to the addition of CPAF substrate WEHD-fmk (100 µM) was added for 30 min. Lysates were incubated for 30 min at 37°C and analysed by Western blotting. Shown is a representative result of three independent experiments. *, non-specific background band. F, CPAF specific cleavage products. (B) CPAF-containing bacterial lysate cleaves CPAF substrates. CPAF-containing E. coli extract was combined with lysates of either vimentin-GFP (vim-GFP) or CK8-myc expressing T-REx-293 cells and incubated for the time points indicated at 37°C. Substrate cleavage was monitored by Western blotting. Untransformed bacteria or E. coli transformed with empty vector were used as controls. Shown is a representative result of three independent experiments. *, non-specific background band. F, CPAF specific cleavage products. (TIF) [file ppat.1002283.s003.tif]

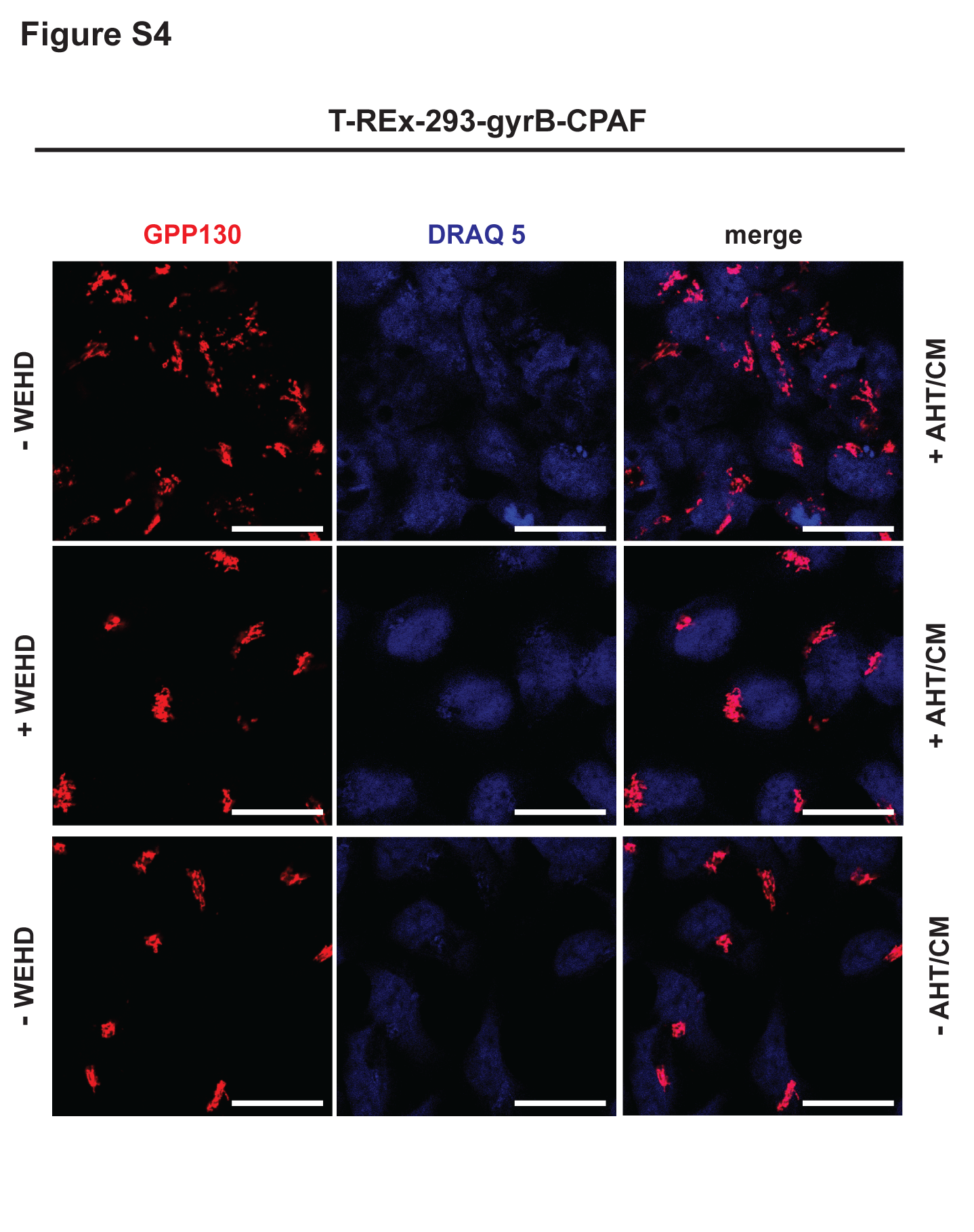

Supplement: Figure S4 — WEHD-fmk inhibits Golgi fragmentation in CPAF expressing cells. CPAF was induced and activated in T-REx-293-gyrB-CPAF cells using AHT/CM (5 h). Another set of cells were left untreated. WEHD-fmk (80 µM) was added at the time of induction (+ WEHD). Cells were fixed and Golgi fragmentation was analysed by GPP130 staining (red channel). Cells were counterstained using the DRAQ 5 (blue channel). Left: GPP130; center: DRAQ 5; right: merged images. Scale bar, 20 µm. (TIF) [file ppat.1002283.s004.tif]

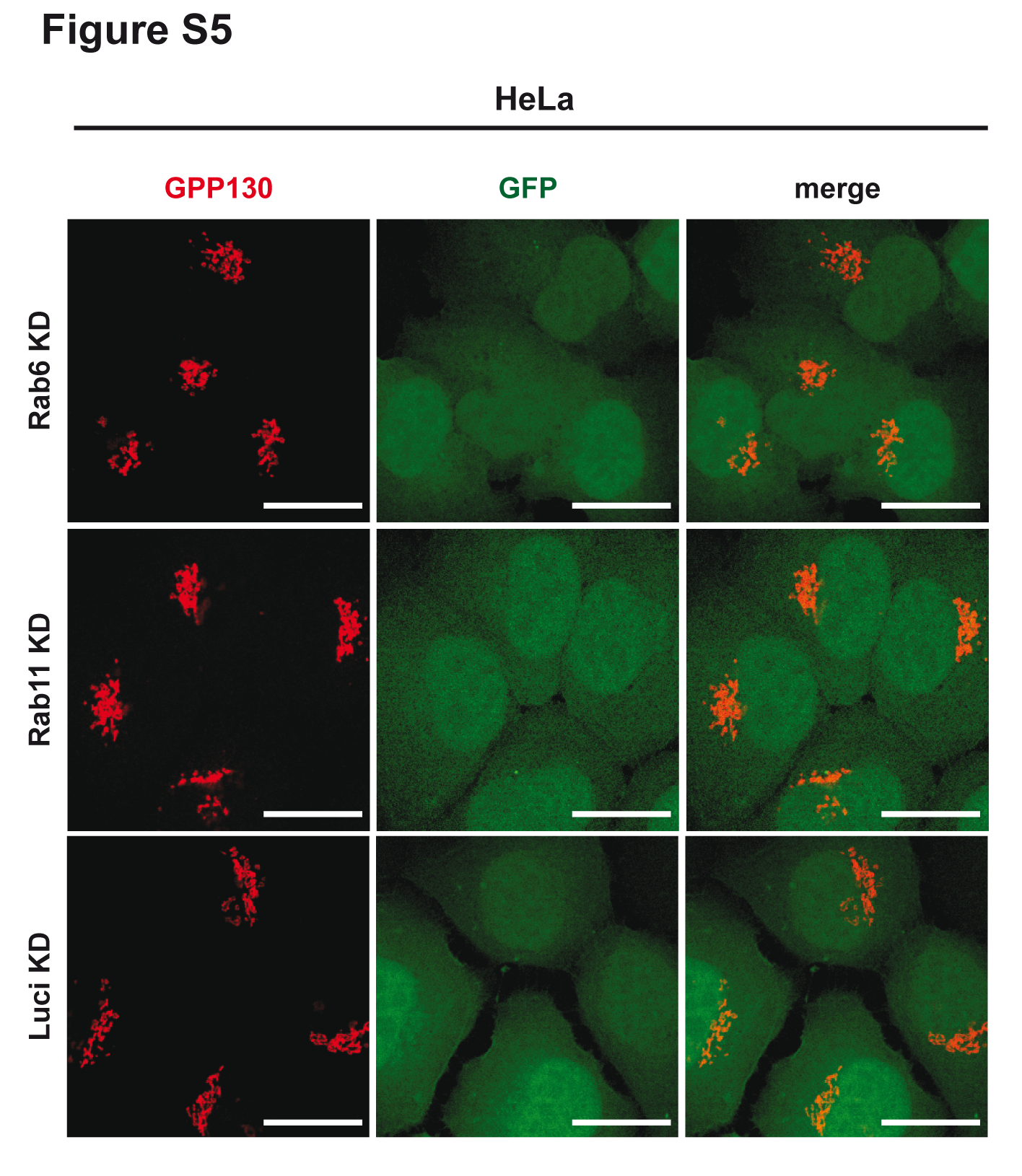

Supplement: Figure S5 — Depletion of Rab6A or Rab11A does not alter GA morphology. HeLa cells were transfected with siRNA specific for Rab6A, Rab11A or Luciferase. 48 h after siRNA transfection, gyrB-CPAF and CMV-eGFP were cotransfected. Cells were fixed and immunostained after an incubation of 48 h. Left: GPP130; middle: GFP; right: merged images. Scale bar, 20 µM. (TIF) [file ppat.1002283.s005.tif]
