# Supplementary material for: Targeting of a Chlamydial Protease Impedes Intracellular Bacterial Growth
Source: PLoS Pathog. 2011 Sep 29;7(9):e1002283. doi: 10.1371/journal.ppat.1002283 (PMC3182938; doi:10.1371/journal.ppat.1002283)
Supplement: Text S1 — Supporting Information: Experimental Procedures. (DOC) [file ppat.1002283.s006.doc]

**Supporting Information: Experimental Procedures**

**Cell lines and culture**

The human embryonic kidney cell line T-REx-293, which stably expresses the tetracycline repressor (Invitrogen, Darmstadt, Germany) was maintained in humidified air at 5% CO_2_ and 37°C in Dulbecco modified Eagle`s minimal essential medium (DMEM) supplemented with 10% fetal calf serum (FCS, tetracycline negative; PAA Laboratories, Pasching, Austria) and 5 µg/µl blasticidin (PAA Laboratories, Pasching, Austria). Stable, gyrB-CPAF expressing T-REx-293 clones were generated by electroporation with the pcDNA4/TO/myc-His-gyrB-CPAF construct and subjected to antibiotic selection as previously reported [1]. The cells were cultured as above but in the presence of zeocin (InvivoGen, San Diego, CA, USA). To induce and activate CPAF 5 ng/µl anhydrotetracycline (AHT; IBA, Göttingen, Germany) and 1 µM coumermycin (CM; Sigma-Aldrich, Steinbach, Germany) were added. HeLa cells were grown in DMEM supplemented with 10% fetal calf serum but lacking blasticidin and zeocin. WEHD-fmk (R&D Systems, Wiesbaden-Nordenstedt, Germany) was added as indicated.

**Chlamydial infection**

The *C. trachomatis* strain (serovar L2) was obtained from the American Type Culture Collection (ATCC). For infection of HeLa cells, the culture medium was replaced with serum free medium without antibiotics and bacteria were added using the specified multiplicity of infection (MOI). Two hours later medium was supplemented with 10% FCS.

**Cell-free proteolysis assay**

As a source of active CPAF, lysate of *E.coli* expressing 6xHis-tagged CPAF or recombinant, purified CPAF (Ni-resin and Source 15Q; GE Healthcare, Uppsala, Sweden) [2] were used. Bacterial lysate or purified protein were mixed with lysate from T-REx-293 cells transiently transfected with a myc-tagged CK8-construct. Reactions were incubated at 37°C for 1 h and subjected to Western blot analysis. In some experiments, a vimentin-GFP fusion construct was used. Reactions were incubated at 37°C and subjected to Western blot analysis. Clasto-lactacystin β-lactone, inhibitors of a Caspase-Family Inhibitor Set (PromoKine, Heidelberg, Germany), WEHD-fmk, VEID-fmk, LEHD-fmk, DEVD-fmk (all four from R&D Systems, Wiesbaden-Nordenstedt, Germany) or z-VAD-fmk (Bachem, Bubendorf, Switzerland) was added 30 min prior to substrate addition.

**Immunoblotting**

Cell extracts were prepared using RIPA-buffer (1% Triton X-100, 0.5% SDS, 0.5% deoxycholate, 1 mM EDTA, 150 mM NaCl, and 50 mM Tris, pH 8.0) supplemented with a protease inhibitor cocktail (Roche, Mannheim, Germany). Proteins were separated by SDS-PAGE and transferred onto nitrocellulose membranes. Antibodies used were directed against GFP, myc (all from Cell Signaling Technology, Beverly, MA, USA) or vimentin (Acris, Herford, Germany).

**RNA interference**

siRNAs were purchased from QIAGEN (QIAGEN, Hilden, Deutschland). HeLa cells were seeded into 12-well plates one day before transfection. Transfection was performed using QIAGEN RNAiFect according to the manufacturer's guidelines. 1 µg siRNA was added to 96 µl Opti-MEM medium (Invitrogen, Darmstadt, Germany), vortexed, mixed with 6 µl RNAiFect and incubated for 15 min at room temperature. The liposome/RNA mix was added to cells with 600 µl growth medium [3]. After 24 h, cells were trypsinised and seeded into new cell culture plates and incubated for another 24 h.

**Immunofluorescence and confocal microscopy**

Cells were seeded onto coverslips and treated as indicated. Cells were then fixed with 2% PFA for 30 min at room temperature. Fixation was followed by treatment with 0.2% Triton X-100 in PBS supplemented with 0.2% BSA. The Golgi marker GPP130 was detected using specific antibodies (Covance, Princeton, NJ, USA). Binding was visualized using fluorescence labeled secondary antibodies. DNA was costained using the fluorescent DNA dye DRAQ 5 (biostatus, Shepshed, Leicestershire, UK) where indicated. In some experiments cells were transfected with a pCMVeGFP plasmid. Cells were mounted in Mowiol. Cells were analysed with an LSCM (Leica TCS SP-1, 63x/1.32 HCX PL APO CS oil lens, Leica Microsystems, Wetzlar, Germany) and images were processed using Adobe Photoshop.

**Light microscopy and analysis of cell morphology**

Changes in cell morphology of T-REx-293-gyrB-CPAF cells were monitored by light microscopy. CPAF was induced for 18 h either in the presence or absence of WEHD-fmk. Images were acquired with an inverted microscope (Axiovert S100, 10x/0.25 lens, AxioCam Hsm Camera, AxioVision software, all from Carl Zeiss AG, Göttingen, Germany).

**Supporting Information: References**

1***.*** Paschen, S.A., J.G. Christian, J. Vier, F. Schmidt, A. Walch, D.M. Ojcius, and G. Hacker. 2008. Cytopathicity of Chlamydia is largely reproduced by expression of a single chlamydial protease. *J Cell Biol* 182:117-127.

2. Huang, Z., Y. Feng, D. Chen, X. Wu, S. Huang, X. Wang, X. Xiao, W. Li, N. Huang, L. Gu, G. Zhong, and J. Chai. 2008. Structural basis for activation and inhibition of the secreted chlamydia protease CPAF. *Cell Host Microbe* 4:52

3. Rejman Lipinski A, Heymann J, Meissner C, Karlas A, Brinkmann V, et al. (2009) Rab6 and Rab11 regulate Chlamydia trachomatis development and golgin-84-dependent Golgi fragmentation. PLoS Pathog 5: e1000615.
